# Enzyme Engineering for In Situ Immobilization

**DOI:** 10.3390/molecules21101370

**Published:** 2016-10-14

**Authors:** Fabian B. H. Rehm, Shuxiong Chen, Bernd H. A. Rehm

**Affiliations:** Institute of Fundamental Sciences, Massey University, Private Bag 11222, Palmerston North 4442, New Zealand; F.Rehm@massey.ac.nz (F.B.H.R.); S.X.Chen@massey.ac.nz (S.C.)

**Keywords:** enzyme, biocatalyst, immobilization, self-assembly, inclusion bodies, protein particles, recombinant enzyme

## Abstract

Enzymes are used as biocatalysts in a vast range of industrial applications. Immobilization of enzymes to solid supports or their self-assembly into insoluble particles enhances their applicability by strongly improving properties such as stability in changing environments, re-usability and applicability in continuous biocatalytic processes. The possibility of co-immobilizing various functionally related enzymes involved in multistep synthesis, conversion or degradation reactions enables the design of multifunctional biocatalyst with enhanced performance compared to their soluble counterparts. This review provides a brief overview of up-to-date in vitro immobilization strategies while focusing on recent advances in enzyme engineering towards in situ self-assembly into insoluble particles. In situ self-assembly approaches include the bioengineering of bacteria to abundantly form enzymatically active inclusion bodies such as enzyme inclusions or enzyme-coated polyhydroxyalkanoate granules. These one-step production strategies for immobilized enzymes avoid prefabrication of the carrier as well as chemical cross-linking or attachment to a support material while the controlled oriented display strongly enhances the fraction of accessible catalytic sites and hence functional enzymes.

## 1. Introduction

Living organisms produce a plethora of enzymes as natural catalysts for numerous synthesis, conversion, or degradation reactions. These biocatalysts are often highly specific and stereoselective in catalyzing a variety of chemical reactions which make them suitable for many industrial applications [1]. Natural enzymes catalyze reactions under mild conditions (e.g., low temperature, atmospheric pressure, and neutral pH) but recent advances in protein engineering enabled production of biocatalysts adapted to a range of non-physiological process conditions such as high temperatures, alkaline, or acidic environments [2]. Industrial applications range from high-volume processes, such as biomass conversion into fermentable sugars for sustainable biofuel production [3,4,5], to high-value low-volume processes, such as medical drug synthesis and biosensors [6,7,8]. The application performance of enzymes in such processes is strongly enhanced by converting the soluble enzymes into insoluble enzyme materials, which aims to increase stability and reusability while physical properties need to be tailored towards process implementation, i.e., the reactor and the process environment [9,10,11]. This review will provide an overview of the existing immobilization strategies such as adsorption, encapsulation, chemical or enzymatic cross-linking to a carrier material, as well as carrier-free approaches where enzymes are cross-linked or self-assembled into insoluble materials. Emphasized will be recent enzyme engineering strategies for in situ production of active insoluble enzymes in a single step.

### 1.1. In Vitro Enzyme Immobilization Strategies

**Figure 1:**
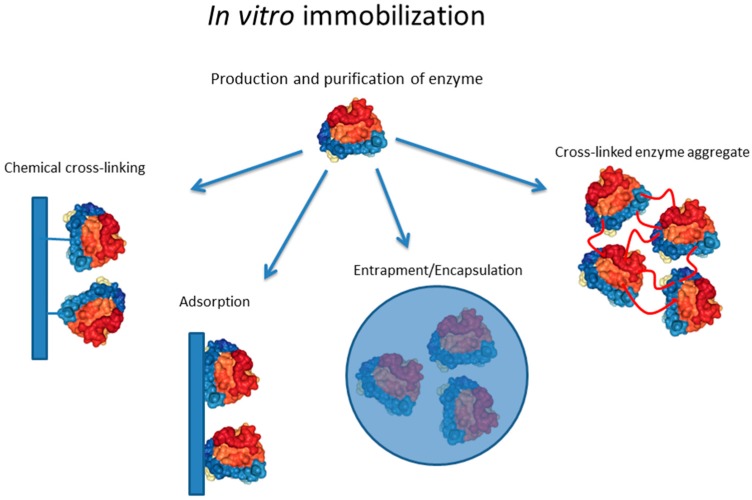
General methods for in vitro immobilization. The crystal structure (1TCC) of lipase B from *Candida antarctica* is depicted.

Immobilization of enzymes to solid supports can be achieved by chemical/enzymatic cross-linking or non-covalent attachment/interaction. The latter mostly utilizes electrostatic, hydrophobic, van der Waals interactions, and hydrogen bonds. The mode of interaction is strongly influenced by the enzyme surface properties and the surface properties of the carrier material, which need to be aligned for proper interaction, i.e., binding of enzyme to carrier. Enzymes themselves cannot only be engineered to show suitable surface properties but can also be engineered to self-assemble in vitro as mediated by an interacting translational fusion partner. Another means of non-covalent immobilization is encapsulation/entrapment where polymers such as alginates self-assemble into spheres in the presence of soluble enzymes. However, if a strong interaction is required, stable covalent bonds need to be introduced. This can be achieved by cross-linking proteins using physicochemical treatment (e.g., alkaline and UV); by using chemical cross-linkers mostly targeting enzyme surface exposed lysine, aspartate, and glutamate residues; or by using specific enzymes, such as sortase, which catalyze transpeptidase reactions. All of the above immobilization strategies require two general processing steps: the production of the biocatalyst and its immobilization (Figure 1).

#### 1.1.1. Materials for Immobilization

Carrier materials for biocatalysts can be composed of inorganic and/or organic matter and their composites can adopt 2D structures such as films and coatings as well as 3D structures such as porous/solid spheres, fibers, and networks. Dimensions of carriers range from nano- to micro-sized structures up to extensive surface areas in high volume bioprocessing. The selection of the carrier material, its shape and dimensions are critical for the application performance of the immobilized biocatalyst under the intended environmental conditions such as temperature, pH, mechanical forces, and viscosity. It should also be noted that processability and surface chemistry of materials are important criteria for carrier selection. This is because the dimension and architecture (spheres, films, etc.) combined with physicochemical surface properties (immobilization mode and functionality) will directly impact applicability. The relevance of various carrier material properties was recently reviewed by Santos et al. [12].

Synthetic polymers such as acrylic resin and nylon [13,14] and natural polymers such as alginates and polyhydroxyalkanoates (PHAs) [15,16] have been used to produce enzyme–carrier assemblies. Carbon nanotubes have been increasingly considered as enzyme carriers in particular for uses in biosensor and bioenergy technologies [17,18]. Inorganic silicates have been successfully used for immobilization of lipases and other technical enzymes [9,19]. Metal particles made of gold, zinc oxide [20], or paramagnetic iron oxide [21,22] were used as enzyme carrier as they enhance enzyme performance given the larger surface-to-volume ratio, high stability, strong adsorption of the target enzyme(s), and electron conductivity. Paramagnetic properties can additionally be harnessed for medical therapeutic and diagnostic (theranostics) applications using magnetic resonance imaging as well as enable fast and simple recovery of the immobilized enzyme using magnets [23,24].

Other common carrier materials include carboxymethyl-cellulose, starch, collagen, agarose, modified sepharose, ion exchange resins, active charcoal, clay, aluminium oxide, titanium, diatomaceous earth, hydroxyapatite, ceramic, celite, or treated porous glass as well as various polymers [25,26]. Materials can be combined to assemble into hierarchical composite structures the properties of which can be fine-tuned towards the targeted reaction conditions [27,28,29].

Porous architectures of carrier materials are preferred as they provide, similar to nanoparticles/nanofibers/nanotubes, a large surface area for efficient high-yield enzyme immobilization and a low diffusion barrier for reactants [5,30].

#### 1.1.2. Chemical and Enzymatic Cross-Linking

Non-covalent cross-linking is often needed for enzyme immobilization in order to avoid leaching of soluble enzyme under various process conditions. In order to suppress leaching, stable covalent bonds are introduced not only to attach the enzyme to a carrier but also to build tight enzyme cages during entrapment as well as to assemble the enzyme into carrier-free cross-linked enzyme aggregates (CLEAs) (Figure 1).

Enzymes naturally contain surface displayed functional groups such as the ε-amino group of lysine, the carboxyl groups of aspartate and glutamate, hydroxyl groups of serine and threonine, and, less frequently, the sulfhydryl group of cysteine. Lysine residues can react with active esters, such as the frequently used *N*-hydroxysuccinimide (NHS) esters, forming stable amide bonds. In aqueous buffers, however, attachment efficiency is reduced due to competing ester hydrolysis. Thus, to achieve greater stability, lysines can be reacted with aldehydes to form a stable secondary amine linkage following reduction with sodium cyanoborohydride. Epoxide-functionalized carriers (diglycidyl ethers) provide targets for nucleophilic attack by amine groups forming less hydrolysis-sensitive bonds [31]. Cysteine residues can form stable thioether bonds with unsaturated carbonyls (e.g., maleimides). Given the low abundance of cysteines in enzymes, they offer the possibility of site-selective cross-linking, in particular by considering site-specific mutagenesis for removal of excess cysteines and insertion cysteines at desired sites [32] (Table 1). The nucleophilic sulfhydryl groups of cysteines can also react with epoxides and NHS esters. The carboxyl groups of aspartate and glutamate can be converted into active esters using carbodiimide which subsequently forms an NHS ester in the presence of NHS. As described above this NHS ester can then react with an amine-bearing carrier [31]. One of the most widely used but less specific cross-linker is glutaraldehyde, which is a bi-functional reagent able to polymerize and to react with a range of chemical groups, primarily involving N-terminal amino groups of proteins but also other groups such as thiols, phenols, and imidazoles [33].

There are also a few enzymes that catalyze peptide bond formation such as transglutaminases, sortases and engineered proteases (e.g., subtiligase), which are applicable for cross-linking enzymes to carriers with active esters or for the assembly of carrier-free CLEAs [34].

#### 1.1.3. Adsorption

Non-covalent binding of a given biocatalyst to a carrier can be achieved by physical adsorption. Whilst this is simple and inexpensive, it is frequently reversible causing leaching of the respective biocatalyst (Figure 1). Hydrophobic enzymes, such as lipases, can be readily adsorbed onto hydrophobic carriers such as macroporous acrylic resins [35]. Ionic interaction of enzyme and carrier follows the principle of ion exchange chromatography where the ionic interaction is dependent on the isoelectric point (pI) of the enzyme, i.e., the pH during adsorption [26]. A specific adsorption approach is realized by affinity binding which harnesses the specific interaction between complementary biomolecules. The strong specificity of the interaction enables oriented immobilization of highly active biocatalysts at high yield [26]. Affinity adsorption is achieved by either coupling the specific enzyme binding domain (e.g., antibody) to the carrier or by genetically fusing a carrier binding domain/motif to the enzyme. This could be a carrier cross-linked antibody that specifically binds to surface epitopes of the enzyme and enables oriented immobilization for improved functionality [36].

#### 1.1.4. Encapsulation/Entrapment

This immobilization approach differs by not using prefabricated support materials, instead assembling the carrier in the presence of the soluble biocatalyst. Materials are required to assemble into supramolecular structures such as gels in the presence of the enzyme (Figure 1). Gel formation conditions need to be compatible with enzyme stability and the resulting gels need to allow efficient diffusion of reactants to enable suitable reaction kinetics. Silica sol-gels prepared by hydrolytic polymerization of tetraethoxysilane are one example. The drying methods strongly impact the pore structure and porosity which are critical morphologies for performance of enzyme–carrier systems [1]. Entrapment of lipases in silica sol-gels increased thermal stability and enhanced reaction rates by several orders of magnitude [19]. Sol-gel entrapment is a cost-effective process suitable for immobilization of a range of enzymes [37,38]. Besides silica sol-gels, synthetic or natural hydrogels are used for entrapment of enzymes. For example, synthetic photo-cross-linkable poly(vinyl alcohol) bearing styrylpyridinium groups (PVA-SbQ) was efficiently applied to entrap various enzymes for bioassay applications [39]. Natural hydrogels, such as agarose, chitosan and alginate have also been used to entrap enzymes. Gelation of these hydrogels is simple and can be conducted at physiological conditions, i.e., preserving functionality of enzymes. However, the ionic cross-linking often does not generate the suitable nanocages required to avoid enzyme leaching. Hence, additional chemical or photo cross-linking is often applied to overcome these caveats [25].

#### 1.1.5. Enzyme Engineering for Immobilization

Recombinant production of biocatalysts offers the opportunity to genetically engineer biocatalyst not only to improve their enzymatic properties but also to enable their targeted site-specific and oriented immobilization to a range of carrier materials. Ultimately, this allows for retaining a high proportion of enzyme functionality by increasing conformational stability and ensuring optimized access of substrate to the immobilized enzyme [26,40]. Furthermore, carriers can be designed to provide specific sites to array different enzymes for generation of multifunctional biocatalysts as required for multistep synthesis, conversion and degradation reactions as well as electron carrier recycling and ATP regeneration reactions. Site-specific immobilization can be non-covalent often harnessing selectivity of specific interactions found in biological systems such as antigen/antibody, avidin/biotin, DNA/protein, polysaccharide/lectin, silaffin/silica and poly-histidine/metal interactions (Table 1). The insertion of charged amino acids into the surface of enzymes enables ionic interaction with charged carrier sites (Section 1.1.3 and Section 1.1.4). Natural or engineered binding domains can be fused to enzymes to mediate specific affinity interaction with the carrier, such as polymers and metals, or enable self-assembly for carrier-free immobilization (Table 1). Indeed, binding motif/domain (e.g., peptides binding inorganic materials, phage display, single-chain variable antibody fragment, and designed ankyrin repeat proteins) libraries can be generated and screened against different carrier materials to isolate specific binders. Self-assembling peptides can be genetically fused to enzymes enabling self-assembly of isolated enzymes under permissive conditions, hence resulting in carrier-free enzyme aggregates [41]. Enzymes can also be chemically modified by adding biomolecules (e.g., biotin, and oligonucleotides) to bind to the cognate ligand (Table 1). A well-established affinity binding mode is based on immobilized anti-enzyme antibodies. Genetic insertion of single specific amino acids displaying functional groups, such as hydroxyl, carboxyl or sulfhydryl groups, provide targets for specific chemical cross-linking to mutually reactive carrier surfaces, hence enabling controlled immobilization for oriented display of the enzyme (Section 1.1.2) (Table 1).

### 1.2. In Situ Enzyme Immobilization Strategies

The aim of enzyme immobilization is often to increase enzymatic stability whilst retaining the desired levels of catalytic activity. Such immobilization may also facilitate extraction from the reaction mixture, further increasing reusability of the biocatalyst. Thus, the overall goal is generally to maximize cost efficiency. From this standpoint, it is clear that reducing the production steps required for the final immobilized biocatalyst would be beneficial. Specifically, it is the requirement for separate enzyme purification and subsequent immobilization steps, as is the case for the aforementioned in vitro enzyme immobilization strategies that imposes a significant limitation on cost-effective use. Moreover, the often harsh and/or toxic conditions of the in vitro strategies limit efficient enzyme immobilization by impacting the structural integrity of the enzyme. With these factors in mind, in vivo enzyme immobilization strategies aim to lower production costs and circumvent any detrimental effects of the in vitro immobilization steps themselves, as will be outlined forthwith.

#### 1.2.1. Formation of Protein Inclusion Bodies

**Figure 2:**
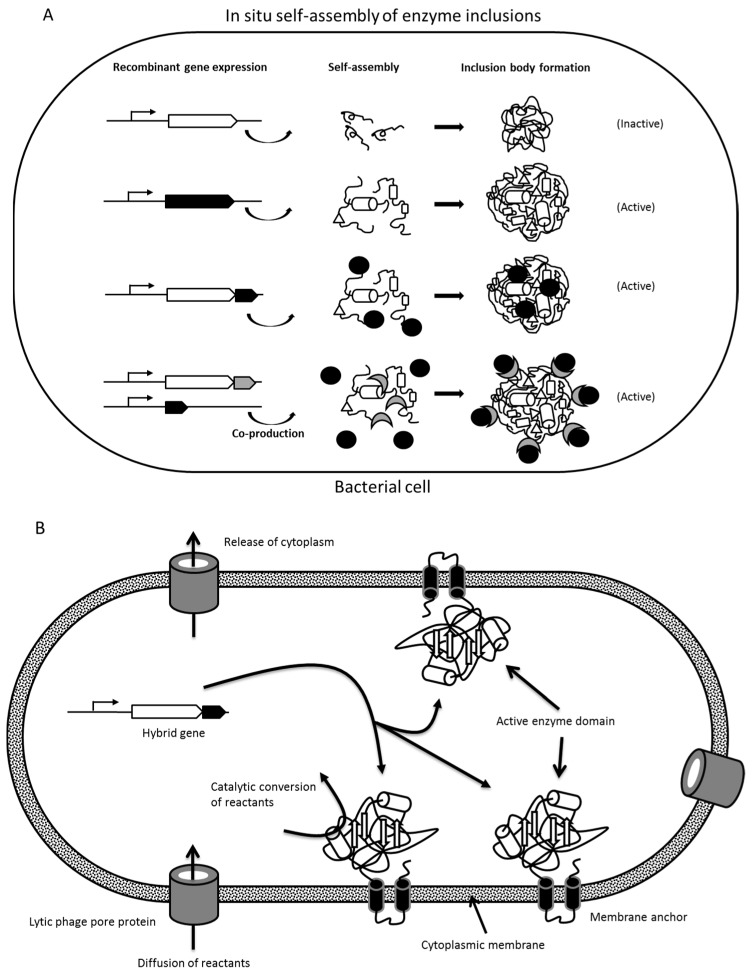
In situ enzyme immobilization: (**A**) Different modes of protein assembly leading enzyme inclusions. Black shapes indicate the target enzyme; (**B**) Immobilization of target enzyme by genetic fusion of a membrane anchor domain. Production of a phage pore enables release of intracellular content while retaining a membrane vesicle with immobilized enzyme.

Overproduction of recombinant proteins causes stress for the production host as it competes with essential native host protein production, impairing yield and quality of the target protein [42,43]. Unlike eukaryotic cells, the bacterial host, such as *Escherichia coli*, has simple protein folding machinery allowing only few post-translational modifications. Bacteria also lack compartmentalization, allowing proteins to be simultaneously synthesized at multiple locations in the cytoplasm [42]. During overproduction of foreign proteins, the protein folding machinery is often overloaded, which can lead to accumulation of protein folding intermediates [42,44]. Some of the folding intermediates that fail to fold into their native conformations will be immediately degraded by the cellular protease mechanisms. However, others can form small proto-aggregates due to various factors, including hydrophobicity, exceeded solubility, and cross-molecular stereospecific interactions [42,45]. During such aggregation, it is predominantly recombinant proteins that are incorporated whilst other non-homologous cellular and recombinant proteins are excluded from this nucleation event [46]. Hence, these bacterial inclusion bodies are pure with low levels of contamination by host cell proteins [47]. In some cases, such inclusion body formation facilitates protein purification as they can be easily separated from cellular debris. However, this often requires laborious refolding to obtain the active protein. Interestingly, correctly folded proteins could also be trapped into the proto-aggregates, the rapid growth of which leads to further aggregation and eventually formation of larger inclusion bodies (usually <1 µm) [42,48] (Figure 2). Therefore, these bacterial protein particles could contain a significant proportion of properly folded and biologically active proteins [42,49]. However, such formation of inclusion bodies containing active proteins, such as enzymes, strongly depends on the individual protein. Interestingly, after the fusion of the proto-aggregates, the spaces between and inside the proto-aggregates are filled in by a cotton-like amorphous matrix, which provides the inclusion bodies with a porous structure allowing diffusion of reactants [42].

The shape and size of inclusion bodies are diverse and often determined by the host organism and the cultivation conditions [50]. Inclusion bodies have been found to exhibit spherical [51], ellipsoidal [52], or cylindrical shapes [53], and are usually 0.05–1 μm in size [47,51,54]. Indeed, protein inclusion bodies may appear spherical in the early stages of cultivation, but, as inclusion bodies continue to grow and reach the bacterial cell wall, they can no longer maintain their spherical shape. It was shown for a few enzymes that they can form active inclusion bodies, hence providing a means of carrier-free enzyme immobilization [55,56].

#### 1.2.2. Insolubility Tags—Self-Assembly of Engineered Enzymes

The inclusion of an appropriate translationally-fused tag in recombinant protein production is commonly used to achieve a greater ease of purification and/or yield of production. However, the fusion of these affinity tags itself can affect the intrinsic properties of the target protein, including solubility, net charge, and folding. For example, a poly-histidine-tag could influence the tertiary structure of target proteins with a hydrophobic C-terminus. Therefore, the addition of the peptide tag fusion partner could possibly impact the folding pathway of the target protein, triggering the self-assembly/aggregation of the respective recombinant protein towards the formation of inclusion bodies [57,58,59] (Figure 2, Table 1). Whilst this is often not desirable in production/purification processes as inclusion bodies are often inactive, the use of selected insolubility tags/fusion partner can promote the formation of defined active aggregates as a means of in situ enzyme immobilization. This is particularly relevant for those enzymes for which overproduction lead to inactive inclusion bodies.

As overproduction of the *Paenibacillus polymyxa* pyruvate oxidase (PoxB) led to formation of active inclusions in *E. coli*, it was fused to the N-terminus of either green fluorescent protein (GFP) or an amylase, which resulted in biologically active inclusion bodies [56]. Hence, PoxB can be considered as a fusion partner promoting the formation of active inclusion bodies in *E. coli*.

In another study, an N-terminally (M)AVTS tetrapeptide-tagged GFP was translationally fused to the N-terminus of the polyester synthase (PhaC). Upon overproduction, this resulted in inclusion body formation of the (M)AVTS-GFP-PhaC fusion protein repressing polyester synthase activity, i.e., the formation of polyester inclusions while retaining structural integrity and functionality of GFP [54]. Therefore, various enzymes were translationally fused to (M)AVTS-GFP-PhaC or only (M)AVTS-GFP as scaffolds to successfully self-assemble enzymatically active inclusion bodies/protein particles [54,60].

#### 1.2.3. Polymer/Lipid Inclusions, Magnetosomes, and Membrane Vesicles

**Figure 3:**
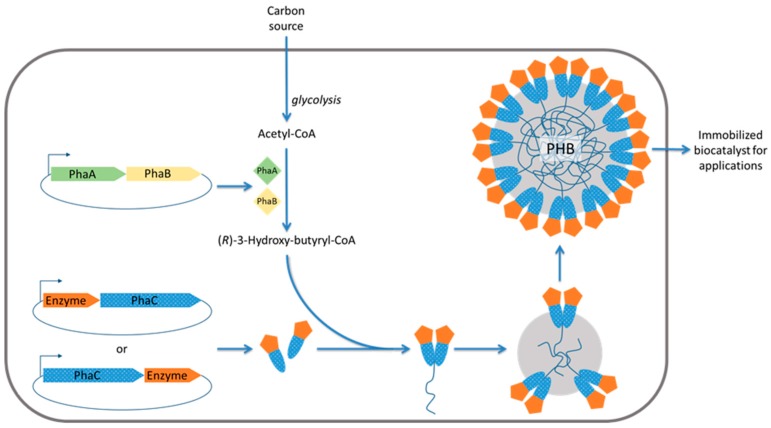
Schematic outlining in situ formation of polyhydroxyalkanoate (PHA) inclusions displaying enzymes. PhaA, β-ketothiolase; PhaB, acetoacetyl-CoA reductase; PhaC, PHA synthase; PHB, polyhydroxybutyrate.

As a means of energy storage under conditions of excess carbon source, various types of lipophilic compounds are deposited as insoluble cytoplasmic inclusions in a range of organisms [61,62]. Inclusions of polyhydroxyalkanoates (PHAs), such as poly(3-hydroxybutyrate) (PHB), are the most common prokaryotic inclusions, but others, such as triacylglycerol (TAG) inclusions, may also be present. Importantly, the hydrophobic core of these inclusions is generally surrounded by an amphiphilic protein shell [63]. Thus, by expressing genes encoding the respective inclusion-associating proteins, or PHA-binding domains, translationally fused to the enzyme desired for immobilization in an inclusion-producing bacterial strain, one-step production of the immobilized biocatalyst was achievable [64]. Engineered PHA inclusions coated with functional protein were recently demonstrated to be applicable for medical applications such as in diagnostics and as vaccine [65] (Figure 3).

In the case of immobilization to PHA inclusions, several fusion partners are available. The most common choice is the PhaC, which remains covalently linked to the inclusion, providing a stronger link than the hydrophobically interacting alternatives [64,66]. One of the first examples of enzyme immobilization to PHA inclusions was demonstrated by genetically fusing PhaC from *Pseudomonas aeruginosa* PAO1 to the *lacZ* gene encoding β-galactosidase, followed by extraction of the enzyme-displaying inclusions [67]. The enzyme beads had high catalytic activity, which diminished much more slowly with increased storage time relative to free β-galactosidase. Continuing on from this, thermostable α-amylase, N-terminally fused to PhaC from *Ralstonia eutropha*, resulted in enzyme beads with similar activity levels as the free counterpart, but with much greater stability at higher temperatures, as would be beneficial for use in starch liquefaction processes [68]. Most recently, lipase B-PhaC fusion beads also revealed increased stability due to immobilization, i.e., activity was stable over a test period of several weeks and the beads were re-usable without diminishing activity [69].

In contrast to PHA inclusions, lipid (e.g., TAG) inclusions do not appear to have any specific, abundantly associated proteins. However, it was demonstrated that the phasin (PhaP1) of *Ralstonia eutropha* H16, which naturally binds PHA inclusions via hydrophobic interactions, is also able to associate with TAG inclusions when heterologously expressed in TAG-accumulating bacteria [70]. Furthermore, translational fusions of PhaP1 with β-galactosidase were demonstrated as binding the lipid inclusions. Potential advantages of immobilization to lipid inclusions over PHA inclusions are yet to be determined.

Magnetosomes are nano-sized lipid/protein coated magnetite (Fe_3_O_4_) or greigite (Fe_3_S_4_) inclusions that are biomineralized by a diverse group of bacteria, the magnetotactic bacteria. Magnetosomes assist bacteria in aligning passively along the geomagnetic field lines. Functional soluble proteins (GFP or luciferase) have been immobilized to the magnetosome surfaces by genetic fusion to anchor proteins specifically targeted to the magnetosome membrane (e.g., MagA, Mms16 or MamC) [71,72]. After cell disruption, the enzyme displaying magnetite particles can be magnetically separated from the cell debris. Their use as enzyme carrier has been reviewed elsewhere [73,74].

In a recent study, the β-galactosidase from *Escherichia coli* K12 was translationally fused by its C-terminus to a membrane anchor derived from rabbit cytochrome b5 which enabled in situ immobilization to the inner surface of the cytosolic membrane [75]. Expression of a gene encoding lytic phage pore protein caused release of the cytosol. The resulting pore-containing membrane vesicles enabled diffusion of reactants and biocatalytic function of the membrane-anchored enzyme.

## 2. Intracellular Self-Assembly towards In Situ Enzyme Immobilization

In situ immobilization strategies have attracted increasing interest as they provide a means for one-step production of already immobilized biocatalyst. This is realized by recombinant production of the enzyme of interest engineered to self-assemble into insoluble aggregates. These aggregates could be, as aforementioned, polyester/lipid inclusions or magnetosomes as well as protein inclusion bodies. There is also the possibility of producing engineered inclusions with binding affinity for biocatalyst co-expressed in the same cell [47,76]. In contrast to in vitro immobilization strategies, the carrier-free in situ approach avoids the immobilization step after isolation of the biocatalyst. In addition, the production of already insoluble biocatalyst within the production host facilitates the isolation of the biocatalyst. After cell disruption, differential centrifugation and/or cross-flow filtration can be applied to enrich/purify the immobilized biocatalyst. In the following sections, we will focus on the two currently most extensively studied approaches of enzyme self-assembly and the in situ attachment of enzymes to PHA inclusions. Both approaches implement the use of bioengineered bacterial production strains.

### 2.1. Enzyme Inclusion Bodies

Overproduction of recombinant proteins in bacteria can result in formation of insoluble aggregates, i.e., inclusion body formation. Often inclusion bodies are inactive improperly folded assemblies of the overproduced foreign protein (Figure 2). This could be the result of oversaturating the protein folding pathway, or lacking folding conditions, such as the need for an oxidative environment or folding-assisting proteins (chaperones). However, in some cases inclusion bodies were demonstrated as retaining their protein function, suggesting functional folding to have occurred. This has been demonstrated in the context of enzymes, such as β-galactosidase, anionic tobacco peroxidase, phosphoenolpyruvate carboxykinase and pyruvate oxidase, [56,77,78,79,80,81]. Importantly, these inclusion bodies still exhibited their corresponding enzymatic activities, thereby suggesting that they contain significant amounts of properly folded proteins [77,78,80,81]. Moreover, these inclusion bodies have a porous surface, which might allow efficient transfer of substrates, permitting interaction with the functional enzyme buried inside [42,49]. Thus, the enzyme activity exhibited by inclusion bodies not only demonstrates the incorporation of functional enzyme into their structures but also emphasizes the potential applications of enzyme inclusion bodies as biologically active catalyst platforms. Self-assembly-based enzyme inclusion bodies represent a novel technique for carrier-free immobilization of biologically active enzyme, and they have been demonstrated as reusable and stable across a range of conditions, which makes them suitable for potential applications in food production and biotechnological industries [49,60,79,81].

### 2.2. Protein Engineering of Enzymes for In Vivo Self-Assembly

To promote biocatalyst self-assembly either overproduction beyond folding capacity or the fusion of a self-assembly promoting peptide/fusion partner is needed.

Galactose oxidase (GOase) was successfully engineered and displayed on the surface of PhaC (polyester synthase) inclusion bodies displaying charged α-helical extensions via the formation of a coiled coil structure [47]. A negatively charged E-coil sequence (EVSALEK)_5_ was fused to the N-terminus of PhaC, and the positively charged K-coil sequence (KVSALKE)_5_ was fused to the C-terminus of GOase. These oppositely charged coil structures are able to form heterodimeric α-helical coiled coil structures which are held together by hydrophobic interactions, generated by a leucine zipper, and by electrostatic interaction between the lysine and glutamate residues of the helical backbone [47]. This heterodimeric α-helical coiled coil was used to bind the target enzyme to the PhaC inclusion bodies when both genes encoding PhaC and GOase were co-produced in the same host organism [47] (Figure 2). Gene expression for these two enzymes was controlled by independently inducible promoters from different plasmids. Isopropyl β-d-1-thiogalactopyranoside was used to induce production of the E-coil-PhaC fusion protein to allow the formation of inclusion bodies. Three hours later, anhydrotetracycline was added to induce the production of soluble GOase-K-coil recombinant protein, followed by overnight cultivation in the presence of the two inducers [47]. The result was that overproduction of E-coil-PhaC fusion alone was able to form inclusion bodies, whilst production of the recombinant GOase-K-coil fusion by itself remained in the soluble fraction where no inclusion bodies could be detected. Co-production of E-coil-PhaC and GOase-K-coil fusion proteins resulted in accumulation of GOase on inclusion bodies. No GOase fusion and only small amounts of PhaC were detected in the soluble fraction, suggesting that GOase is almost completely captured by the PhaC inclusion bodies in the cytoplasm via the heterodimeric α-helical coiled coil structure [47]. A similar technique has also been used to co-display alcohol dehydrogenases and formate dehydrogenase on the surface of PhaC inclusion bodies [47,82].

In another study, PhaC served as a self-assembly promoting fusion partner [54]. Initially, GFP was used to monitor functionality of protein particles for which self-assembly was promoted by fusion to an inactive PhaC variant [54]. Fluorescence of particles would suggest structural integrity and functionality of GFP. Indeed, overproduction of only GFP did not lead to formation of GFP protein particles. Active PhaC mediates the self-assembly of polyester inclusions, however, this requires the presence of the enzymes PhaA and PhaB, which catalyze conversion of acetyl-CoA to *R*-3-hydroxybutyryl-CoA, the precursor molecule for polyester synthesis [54] (Figure 2). Interestingly, upon adding the N-terminal peptide tag (M)AVTS to a GFP-PhaC fusion the formation protein/polyester hybrid particles was observed when PhaA and PhaB were co-produced (Figure 2B). In the absence of PhaA and PhaB, however, overproduction of (M)AVTS-GFP-PhaC resulted in the formation of only fluorescent protein (FP) particles. The same result was obtained by using an inactive variant of PhaC independent of PhaA and PhaB. PhaC was inactivated by replacing the catalytic nucleophile cysteine residue 319 with alanine [54]. It was therefore concluded that (M)AVTS-GFP protein particle formation was not mediated by small polyester inclusions as it was independent of PhaC activity [54].

Further investigations showed that inactive PhaC (C319A) in the (M)AVTS-GFP fusion could be replaced by other proteins, such as *N*-acetyl-d-neuraminic acid aldolase (NanA) without compromising the formation of GFP protein particles [54]. Thus, PhaC (C319A) might act as a larger linker between GFP and the target proteins, and indeed, the formation of GFP protein particles required overexpression of a hybrid encoding the (M)AVTS-fluorescent protein with a larger C-terminal extension (e.g., PhaC) prior to fusion of the protein of interest in order to form functional FP particles [54]. Accordingly, various FP particles could be spontaneously formed in a manner similar to GFP by using other types of FPs, including enhanced yellow fluorescent protein (EYFP), enhanced cyan fluorescent protein (ECFP), and far-red protein HcRed (HcR) [54]. These FPs were N-terminally extended with (M)AVTS which mediated self-assembly of their respective FP particles when fused to the N-terminus of a larger linker, including the inactive PhaC (C319A) or NanA [54].

Since these FP particles were produced at high yield inside genetically engineered *E. coli* amounting to ~30% of cellular dry weight, they were considered for enzyme immobilization. The protein of interest was replaced with the α-amylase from *Bacillus licheniformis*, NanA from *E. coli*, and organophosphohydrolase (OpdA) from *Agrobacterium radiobacter*, respectively. These enzyme-displaying FP particles were highly active and stable across a wide range of temperatures, pHs, and different storage conditions [60]. Moreover, these immobilized enzymes can be recycled and reused without loss of performance [60]. It should be noted that the tested enzymes exhibited different quaternary structures such as the monomers (α-amylase), dimers (OpdA) and tetramers (NanA) further supporting the versatility of this in situ immobilization approach.

The aforementioned (Section 2.1) possibility that certain enzymes/proteins form active inclusions bodies offer the opportunity to employ these proteins as scaffolds for incorporation of the enzyme of interest. For example the pyruvate oxidase forms active inclusion bodies within *E. coli* and was successfully used as fusion partner for an amylase (enzyme of interest) to form inclusion bodies with amylase activity [56].

### 2.3. Engineered Polyhydroxyalkanoate Inclusions for Enzyme Display

Polyhydroxyalkanoates represent a group of bioplastics/biopolyesters consisting of *R*-3-hydroxy fatty acids. Biologically, they serve as energy/carbon stockpiles for bacteria or archaea under high carbon, yet low nutrient, conditions. They are deposited in a given organism’s cytoplasm as water-insoluble, spherical inclusion bodies (granules) ranging from 50 to 500 nm in diameter [83]. Although some details of their structure remain to be elucidated, PHA, often poly(3-hydroxybutyrate), is the major granule constituent, comprising the large, amorphous, hydrophobic granule core surrounded by attached or embedded proteins which make up the ~4 nm boundary layer [83]. These so-called granule-associated proteins (GAPs) have been primarily characterized in the context of the model organism *Ralstonia eutropha* and include the PHA synthases (PhaC), depolymerases (PhaZ), as well as regulatory (PhaR) and structural proteins (phasins, PhaP).

PhaC, the central enzyme of PHA biosynthesis, stereoselectively catalyzes the conversion of *R*-3-hydroxyacyl CoA thioesters to PHA with concomitant CoA release [84]. This requires prior steps for synthesis of the precursor: the β-ketothiolase (PhaA) catalyzes the condensation of acetyl-CoA and the acetoacetyl-CoA reductase (PhaB) catalyzes the subsequent reduction [85,86]. Driven by the diverse material properties of PHAs in combination with their biodegradability and biocompatibility, this biosynthesis pathway has been recombinantly established in industrial bacterial production hosts better suited for production, such as *E. coli*, for a variety of medical and industrial applications. However, the use of extracted PHA granules themselves, rather than extracting and further processing the polymer, has only recently emerged. In addition to exploiting the spherical nature of these granules, strategies towards oriented enzyme immobilization to their surface have primarily made use of the covalent attachment of PhaC directly to the granule, thus circumventing the cost, toxicity, and orientation issues associated with chemical crosslinking steps [16]. The general approach has been to produce PhaA and PhaB as soluble proteins and translationally fuse the enzyme of interest to PhaC under the inducible control of a T7 promoter. Subsequently, extraction and purification results in enzymatically active PHA beads, the properties of which have been demonstrated as superior to their soluble enzyme counterparts in the context of a range of potential applications. It is worth mentioning that these PHA beads have also been demonstrated as enabling the display of non-enzyme protein functions, such as antigens for medical application including vaccines and diagnostics [65].

PhaC has been divided into four classes (I–IV) on the basis of quaternary structure and composition of the synthesized PHA. Whilst a Class II PhaC, which yields medium-chain-length PHAs (C_6_–C_14_), from *P. aeruginosa* was used for the initial demonstration of β-galactosidase immobilization [67], subsequent studies switched to using Class I, which yields short-chain-length PHAs (C_3_–C_5_). This allowed easier metabolic engineering towards precursor supply from acetyl-CoA while being able to use a range of carbon sources. In this context, a range of immobilized enzymes of a variety of molecular weights and quaternary structures have demonstrated the versatility of this immobilization platform [7,68,69,87,88,89]. However, enzyme immobilization using PhaP, instead of PhaC, as the fusion partner has also been demonstrated. One example used human tissue plasminogen activator (rPA) fused to PhaP via a thrombin cleavage site-containing linker [90]. This resulted in active beads as well as active enzyme in solution following cleavage. However, as PhaP only hydrophobically interacts with the granule, the covalent attachment of PhaC fusions is more suitable for harsh reaction conditions and reuse over multiple cycles.

Importantly, the orientation of the immobilized enzyme is dictated by how it is fused to PhaC (N-terminally or C-terminally). Conceivably, depending on the enzyme, this could greatly impact substrate accessibility and therefore enzymatic activity. This was demonstrated in a study on *N*-acetylneuraminic acid aldolase which performed better when fused to the C-terminus [7]. Thus, fusion to each terminus should ideally be assessed and compared.

Several desirable properties have been achieved by PHA-based enzyme immobilization. Immobilized α-amylase had a Michaelis-Menten constant (K_m_) of 5 µM and a maximum turnover rate (V_max_) of 506 mU/mg protein which is consistent with that of free α-amylase. Moreover, the enzyme was shown to remain stable at 85 °C and retained 78% of its activity following three reaction cycles. Immobilized organophosphohydrolase (OpdA) further demonstrated such stability, despite reduced activity relative to free OpdA [88]. The apparent melting temperature increased by 1.58% and a slow decline in activity was measured from 35–80 °C, in contrast to the rapid decline of the free form. Additionally, long term storage (five months) in tap water at 25°C showed that 15% of activity was still retained with the immobilized form. Even more impressive levels of stability were demonstrated with immobilized N-ethylmaleimide reductase (NemA) which saw no reduction in enzyme activity after storage for nine months at 4°C and had a K_m_ for NADH (nicotinamide adenine dinucleotide) similar to the free form. Similarly, immobilized lipase B, which contains several disulfide bonds, showed stable activity over 50 days of storage [69]. These findings demonstrate a diverse range of enzymes as having enhanced stability yet similar levels of activity relative to their free counterpart. Thus, the cost-efficient one-step production of the beads combined with their reusability and increased storage stability could be greatly economically beneficial to several enzyme-catalyzed bioprocesses.

### 2.4. Uses of In Situ Immobilized Enzymes

Various biocatalysts have been successfully immobilized by using in situ immobilization techniques. Carrier-free enzyme particles self-assembled inside the bacterial production host can be produced at high yields and can serve as a versatile platform for enzyme immobilization suitable for a variety of applications. Protein particles are made of natural components and hence are biodegradable. There is no chemical cross-linking as the enzyme-bearing components are tightly connected via non-covalent bonds inherently suggesting some degree of instability due to the possibility of disassembly. To our knowledge there is no related product being used in an industrial process or being commercialized. However, the suitability of aforementioned FPs (Section 2.2) in enzyme immobilization for industrial uses was recently elaborated in more detail [60]. The following enzymes were tested: thermostable α-amylase from *Bacillus licheniformis*, NanA from *Escherichia coli*, and OpdA from *Agrobacterium radiobacter*. Respective genetic fusions led to FP particles which could be isolated and stably maintained outside the cell while exhibiting the specific enzyme activity. These enzyme-FP particles were stable across a range of temperatures, pH, and storage conditions and were demonstrated as reusable. For example, the thermostable α-amylase-FP particles remained active after incubation at 4–85 °C and at pH 4–10. Shelf life studies suggested stability for at least three months at 4 °C. Amylase, NanA, and OpdA particles were proposed for applications in biomass conversion (starch liquefaction), pharmaceutical production (sialic acid synthesis), and bioremediation (insecticide degradation), respectively.

Other protein inclusion-based uses include the immobilization of the K-coil tagged GOase to E-coil displaying PhaC inclusion bodies which were proposed as cost-effective means for enzyme display [47]. Furthermore, the simple overproduction of β-galactosidase alone leads to the formation of functional inclusion bodies [81]. Immobilized GOase and β-galactosidase were proposed for uses in food processing.

The most extensively studied strategy of in situ immobilization is genetic fusion of PhaC with the enzyme(s) of interest [66]. When overproduced in PHA precursor providing hosts, PHA inclusions densely coated with covalently linked and oriented enzymes can be obtained at high yield and functionality [16]. The PHA core is the natural carrier and PhaC the anchor dictating the orientation of the fusion partner enzyme via defined interaction with the core. A range of industrially relevant enzymes varying in tertiary and quaternary structure, including secreted and non-secreted enzymes, have been successfully immobilized using this PHA inclusion approach. In addition, it was possible to genetically fuse up to three enzymes to PhaC enabling production of PHA inclusion for multistep catalysis such as the synthesis of antiviral sialic acid from *N*-acetyl-glucosamine [7,91]. Enzyme-displaying PHA beads are biodegradable and can be applied in bioremediation in the polluted environment where the activity of the degrading enzymes, such as OpdA, is temporarily stabilized but ultimately exposed to natural biodegradation. A thermostable amylase immobilized to PHA inclusions enabled to assess their structural integrity and stability particularly with respect to high temperatures. Results suggested that the PHA inclusions were resistant to high temperature treatment (>5 h at 85 °C) retaining enzyme function and PHA inclusion structure. The PHA inclusion based enzyme immobilization platform offers versatility and stability combined with high functionality due to oriented display and a large surface-to-volume ratio. These nano-/micro-beads offer an economically interesting enzyme immobilization strategy with potential applicability in a range process conditions including natural environments.

## 3. Conclusions and Prospects

Enzyme engineering has been extensively applied to improve biocatalysts by enhancing their process-related properties using random mutagenesis approaches to generate variant libraries, which are subsequently subjected to screening for the best performers. Increasing knowledge about enzyme structure–function relationships has also enabled rational design approaches to develop variants with advantageous properties. Accordingly, genetic engineering also enables the development of enzymes inherently able to bind to various carrier materials or to facilitate adsorption/cross-linking to carrier materials for oriented and more efficient immobilization, providing enhanced stability and functionality. While these approaches are promising, new strategies to reduce production costs and to increase functionality of immobilized biocatalysts are in demand. In this context, in situ immobilization strategies are emerging as a cost-effective one-step production alternative of immobilized enzymes. Generally, this involves the engineering of enzymes to self-assemble into nano-/microsized supramolecular structures inside the cell without the need of prefabrication of a carrier and subsequent attachment of the biocatalyst. This new approach offers the potential of cost-effective manufacture of biocatalysts, which are produced as already immobilized. The immobilized and insoluble nature of the produced biocatalyst also facilitates downstream processing, i.e., the efficient recovery of the biocatalysts by such as centrifugation and/or filtration. Various in situ immobilization strategies have recently been explored. A promising and more extensively studied approach recruits the bacterial machinery for production of insoluble polyester (PHA) inclusions. Genetic fusion of the polyester synthase with the enzyme(s) of interest mediate prolific production of stable PHA beads displaying the respective enzyme in a highly functional oriented mode covalently linked to the PHA core. Further investigations will be needed to demonstrate the implementation and applicability of the various biocatalysts immobilized using in situ immobilization strategies.

## Figures and Tables

**Table 1:** Enzyme engineering towards site-specific immobilization.

Enzyme Engineering	Carrier Type/Site	Mode of Interaction
*Genetic engineering of insertion or fusion:* Poly-Histidine (6 or more) Peptides/Epitopes: ○ Flag ○ Myc ○ HA ○ Strep ○ LPXTG ○ SNAP * ○ CLIP * ○ HaloTag ○ GEPI ○ SAP Binding domains ○ Chitin ○ Cellulose ○ Maltose ○ PHA ○ DNA Amino acids/functional groups ○ Lysine ○ Cysteine ○ Aspartate ○ Glutamate ○ Poly-Lysine ○ Poly-Aspartate ○ Poly-Glutamate	Immobilized metal ion ○ Monoclonal antibody ○ Monoclonal antibody ○ Monoclonal antibody ○ Engineered avidin ○ Diglycine-surface ○ Benzylguanine-surface ○ Benzylguanine-surface ○ Chloroalkane-surface ○ Inorganic carrier ○ Carrier-free self-assembly ○ Chitin ○ Cellulose ○ Maltose-surface ○ PHA ○ DNA ○ NHS-surface ○ Maleimide-surface ○ Amine-modified surface ○ Amine-modified surface ○ Negatively charged surface ○ Positively charged surface ○ Positively charged surface	Metal coordination ○ Affinity ○ Affinity ○ Affinity ○ Affinity ○ Covalent ○ Covalent ○ Covalent ○ Covalent ○ Non-covalent ○ Non-covalent ○ Affinity ○ Affinity ○ Affinity ○ Hydrophobic ○ Watson-Crick base pairs ○ Covalent ○ Covalent ○ Covalent ○ Covalent ○ Ionic ○ Ionic ○ Ionic
*Chemical modifications:* Biotinylation DNA	Avidin/streptavidin Complimentary DNA or positive charges	Affinity Watson-Crick base pair or ionic
*Enzymatic modifications:* Biotin (biotin ligase) Peptides (sortase A) Farnesyl azides or alkynes (PFT) Primary amino-containing probes (TG) Alkyl or aryl azido-lipoic acids (Lpl)	Avidin coated surface Diglycine-functionalized glass “click” chemistry functional group displaying surface DNA displaying surface “click” chemistry functional group displaying surface	Affinity Covalent Covalent Watson-Crick base pairs Covalent

* Self-labelling tags (~20–33 kDa); Flag, peptide with sequence DYKDDDDK; Myc, peptide with sequence EQKLISEEDL; HA, Haemagglutinin; Strep, peptide with sequence WSHPQFEK; LPXTG, sortase recognition motif peptide with sequence LPXTG where X is any amino acid; SNAP, 19.4 kDa polypeptide; CLIP, a modified SNAP; HaloTag, modified haloalkane dehalogenase designed to covalently bind to synthetic ligands; GEPI, genetically modified peptides binding inorganics (there are also natural peptide binding inorganics such as silaffins binding silica); SAP, self-assembling peptides; PHA, polyhydroxyalkanoates; PFT, protein farnesyl transferase; TG, transglutaminase; Lpl, lipoic acid ligase.
